# A case report of a family with developmental arrest of human prokaryotic stage zygote

**DOI:** 10.3389/fcell.2024.1280797

**Published:** 2024-03-28

**Authors:** Tianzhong Ma, Songxia Zhou, Xuezhen Xie, Jingyao Chen, Jing Wang, Guohong Zhang

**Affiliations:** ^1^ Reproductive Medicine Center, Affiliated Hospital of Guangdong Medical University, Zhanjiang, Guangdong, China; ^2^ Department of Pathology, Shantou University Medical College, Shantou, Guangdong, China

**Keywords:** pronuclear arrest, zygote, *RGS12*, Ca^2+^ oscillation, case report

## Abstract

To study the genetic variation leading to the arrest phenotype of pronuclear (PN) zygotes. We recruited a family characterized by recurrent PN arrest during *in vitro* fertilization (IVF) and intracytoplasmic sperm injection cycles (ICSI) and performed whole-exome sequencing for 2 individuals. The transcriptome profiles of PN-arrest zygotes were assessed by single-cell RNA sequencing analysis. The variants were then validated by PCR amplification and Sanger sequencing in the affected individuals and other family members. A family characterized by recurrent PN arrest during IVF and ICSI cycles were enrolled after giving written informed consent. Peripheral blood samples were taken for DNA extraction. Three PN-arrest zygotes from patient III-3 were used for single-cell RNA-seq as described. This phenotype was reproduced after multiple cycles of egg retrieval and after trying different fertilization methods and multiple ovulation regimens. The mutant genes of whole exon sequencing were screened and verified. The missense variant c. C1630T (p.R544W) in *RGS12* was responsible for a phenotype characterized by paternal transmission. *RGS12* controls Ca^2+^ oscillation, which is required for oocyte activation after fertilization. Single-cell transcriptome profiling of PN-arrest zygotes revealed defective established translation, RNA processing and cell cycle, which explained the failure of complete oocyte activation. Furthermore, we identified proximal genes involved in Ca^2+^ oscillation–cytostatic factor–anaphase-promoting complex (Ca^2+^ oscillation–CSF–APC) signaling, including upregulated *CaMKII*, *ORAI1, CDC20,* and *CDH1* and downregulated *EMI1* and *BUB3*. The findings indicate abnormal spontaneous Ca^2+^ oscillations leading to oocytes with prolonged low CSF level and high APC level, which resulted in defective nuclear envelope breakdown and DNA replication. We have identified an RGS12 variant as the potential cause of female infertility characterized by arrest at the PN stage during multiple IVF and ICSI.

## Background

*In vitro* fertilization (IVF) is now routine for treating infertile women and has brought at least 8 million babies into the world. Indeed, the earliest embryonic development after fertilization is a complex process, including the formation of spermatozoa and oocyte pronuclei (two-pronuclear [2PN] zygote), cytoskeletal rearrangement, singamy, and initiation of cleavage of the zygote. After IVF trials, about 5% of fertilized human oocytes present early developmental arrest at the PN stage (Schmiady et al., 1987). Homozygous mutations in *TLE6* (MIM: 612399) and *PADI6* (MIM: 10363) have been reported to cause embryonic arrest at the 2- to 4-cell stage with normal cleavage in consanguineous families (Alazami et al., 2015; Xu et al., 2016). A patient presenting complete cleavage failure in 2PN oocytes after IVF carried the homozygous mutation c.322G > A (p.Glu108Lys) in *TUBB8* (Yuan et al., 2018). However, the crucial gene responsible for PN-arrest zygotes from familial individuals remains largely unknown.

Oocyte activation is Ca^2+^-dependent, characterized by an increase in and spreading of intracellular Ca^2+^ waves. After formation of the male and female pronuclei, calcium signaling continues to play a crucial role in 2PN fusion, DNA synthesis, and initiation of the first cleavage. Therefore, optimal Ca^2+^ oscillation is required for both oocyte activation and 2PN fusion. Chemical artificial oocyte activation with the A23187 Ca^2+^ ionophore improved embryo development to the cleavage stage for zygotes with arrest at the PN stage. However, artificial oocyte activation with a Ca^2+^ ionophore resulted in partial success (Darwish and Magdi, 2015; Xi et al., 2020), so defects in an unknown mechanism can also contribute to Ca^2+^ oscillations causing 2PN fusion failure.

Here, we performed whole exome sequencing (WES) and single-cell RNA sequencing (RNA-seq) in a family characterized by recurrent PN arrest during IVF and intracytoplasmic sperm injection cycles. Our analysis implicated the causative variant in *RGS12*, a gene that controls Ca^2+^ oscillation, which is required for oocyte activation after fertilization.

## Material and methods

### Ethics approval and consent to participate

This study was approved by the Ethics Committee of Guangdong Medical University Affiliated Hospital (YS2018010), and written informed consent was obtained from participants. Patients gave written informed consent for the use of abandoned zygotes and peripheral blood for research on the arrest mechanism of PN stage, with no monetary payment. All procedures used were performed in accordance with the relevant guidelines and regulations.

### Family recruitment

Families were recruited in the Reproductive Medicine Center at the Affiliated Hospital of Guangdong Medical University based on the observation of PN arrest during regular IVF treatment of 2 siblings. Eligible families and controls were enrolled after giving written informed consent. Peripheral blood samples were taken for DNA extraction.

### Patients, ovarian stimulation, oocyte retrieval, and IVF/intracytoplasmic sperm injection (ICSI) procedures

Females III-2 and III-3 were 29 and 25 years old, with BMI of 18.82 and 18.92 kg/m^2^. They both had primary infertility due to unexplained infertility. In the family, the women’s ovarian function and sex hormones were normal, and the men’s semen was normal. The peripheral blood chromosomes of both woman and man were normal as well (Supplementary Table S1).

IVF and ICSI were performed according to the laboratory routine insemination procedures on the day of oocyte retrieval (Day 0). The presence of 2 pronuclei (2PN) was observed 16–18 h after insemination or injection, then zygotes were cultured in 25 µL pre-equilibrated cleavage medium at 37°C under 6% CO_2_. Embryo morphology was evaluated at 42–46 h (Day 2) and 68–72 h (Day 3) after insemination. Male and female pronuclei that continued to separate on Day 2 and 3 without fusion were defined as PN-arrest zygotes. More details of IVF/ICSI procedures are given in the following.

#### Ovarian stimulation

The ovulation promotion scheme and embryo development of patients with different egg retrieval cycles are described in detail in Supplementary Table S2.

Long agonist protocol: Briefly, 5–7 days after ovulation or after 15 days of being on pre-IVF Marvelon (N.V. Organon, Netherlands), gonadotropin-releasing hormone analogue (GnRH-a; single dose of 0.8–1.875  mg; Decapepty, Ferring, Germany) was administered. The GnRH-a administration phase ended on the human chorionic gonadotropin (hCG) administration day. The Gn phase of therapy, which started 14 days after starting GnRH-a delivery and finished on the hCG injection day, included a natural or synthetic follicle stimulating hormone (FSH) preparation (dose Gonal-F, Merck Serono, Germany), a luteinizing hormone (LH) preparation (dose Luveris, Merck Serono, Germany), and, finally, hCG (10,000 IU, Livzon Pharmaceutical Group, China). Eggs were harvested 34–36 h after hCG injection (generally, a single follicle with diameter of up to 19 mm, 2 follicles with diameter up to 18 mm, or 3 follicles with diameter up to 17 mm), when E2 levels were monitored (generally reaching a mean of 250–300 ng/L per dominant follicle [≥16 mm], and a proportion of dominant follicles as high as 60%).

Mild stimulation protocol: Without GnRH-a downregulation, a small number of ovulation-stimulating drugs is usually applied from the first 3–5 days of menstruation. Simple Gn was used to promote ovulation, or Gn was combined with oral ovulation-stimulating drugs to promote follicle growth After follicles grew and matured, after giving the hCG trigger, the egg was retrieved the next day.

#### Sperm preparation

Ejaculated samples were obtained after 3–5 days of abstinence. Semen was screened by the direct upstream method of fertilization culture fluid (G-IVF, Vitrolife Sweden AB). The pellet was resuspended in 0.3 mL fertilization culture fluid.

#### Oocyte retrieval

Transvaginal follicular aspiration was performed 36 h after recombinant hCG administration. After cumulus oocyte complexes were retrieved in 1 mL modified human tubal fluid medium (G-MOPS, Vitrolife Sweden AB) at 37°C, they were cultured in cleavage medium (G-1, Vitrolife Sweden AB) covered with light mineral oil (Vitrolife Sweden AB) at 37°C in 6% CO_2_ for 2–4 h before conventional IVF, ICSI or IVF + ICSI (half the eggs were fertilized as routine and half by microinjection).

#### Oocyte preparation and insemination

All cumulus oocyte complexes were enzymatically treated with 80 µL hyaluronidase (Vitrolife Sweden AB) for 30–60 s with the aid of mechanical denudation to remove cumulus cells. The denuded oocytes were examined for maturity and integrity. Only metaphase II (MII) oocytes that had extruded the first polar body were injected by spermatozoa.

#### Zygote and embryo assessment

Fertilization was assessed 16–18 h after IVF or ICSI. On day 3 (68–72 h), cleaved embryos were morphologically graded by evaluating cell number, size, fragmentation and nucleation.

### Whole-exome sequencing (WES) and data

WES was performed for 2 affected individuals and 3 unaffected individuals, including an unaffected sibling and parents. Germline genomic DNA was subjected to exome capture (60 Mb) with the Agilent SureSelect Human All ExonV6 kit according to the manufacturer’s instructions (Agilent, Santa Clara, CA). Paired-end sequencing, resulting in 150 bases from each end of the fragments, involved using a HiSeq PE150 Genome Analyzer (Illumina) at Novogene Bioinformatics Technology (Beijing). Sequencing reads were mapped to the reference genome (GRCh37, UCSC hg19) by using the Burrow-Wheller Aligner and were analyzed by using the Genome Analysis Toolkit (GATK, v3.1) for calling single nucleotide variants, insertions and deletions. The 1,000 Genomes, Exome Sequencing Project (ESP6500), Exome Aggregation Consortium (ExAC) and an in-house database were used to annotate the minor allele frequency for each variant. *In silico* analysis, Sort Intolerant from Tolerant (SIFT), Polymorphism Phenotyping v2 (PolyPhen-2), MutationAssessor, and Genomic Evolutionary Rate Profiling (GERP++) were used to predict the impact of each non-synonymous variant.

### Variant filtering

The pipeline was designed to filter heterozygous variants 1) shared by both affected individuals; 2) absent in other unaffected family members; 3) not previously reported or reported to have a frequency <0.1% in the public databases 1,000 Genomes, ESP6500, and ExAC and the in-house database; and 4) frameshift, nonsense, splice-site and missense variants predicted to be damaging in at least 3 of the 4 algorithms SIFT, PolyPhen-2, MutationAssessor, and GERP++.

### Sanger sequencing validation and segregation analysis for candidate variants

The variants were then validated by PCR amplification and Sanger sequencing in the affected individuals and other family members. The *RGS12* gene-specific primers to generate the variation were 5′-CAG​GTT​CTG​GGA​CCT​AAA​CAA​G-3′ (forward) and 5′-GAC​TGT​GCA​AGC​TGG​TGA​CT-3′ (reverse). Variants were evaluated for co-segregation based on an autosomal-dominant mode of inheritance.

### RNA library preparation and RNA sequencing (RNA-seq)

Three PN-arrest zygotes from patient III-3 were used for single-cell RNA-seq as described (Zhang et al., 2018). Briefly, zygotes were transferred into lysate buffer by using a mouth pipette, and whole-cell lysates underwent reverse transcription reaction according to the manufacturer’s instructions. Terminal deoxynucleotidyl transferase was used to add a poly(A) tail to the 3ʹ end of the first-strand cDNA, then 20 + 10 cycles of PCR were used to amplify the single-cell cDNA. The libraries were sequenced on the HiSeq PE150 Genome Analyzer platform at Annoroad Gene Technology (Beijing; http://www.annoroad.com).

### Transcript alignment and assembly

Overall read quality was checked by using FASTQC v.0.11.5. The raw sequence data, in the form of FASTQ files, were aligned to the human genome (GRCh38, Ensembl Homo_sapiens) by using HISAT2 (v. 2.1.0) and SAMTOOLS (v1.3.1). Read count and Fragments Per Kilobase Million (FPKM) mapped reads for each sample were generated by using HTSeq v0.6.0.

### Differential expression analysis

HTSeq read counts were uploaded into RNA-seq 2G (http://52.90.192.24:3838/rnaseq2g/) for DESeq2 analysis. Normalization involved default settings (“normalize count by DESeq/normalize logged by Loess”). P-values were adjusted with the Benjamini and Hochberg method for controlling the false discovery rate (FDR). Genes with FDR *p* < 0.05 and fold change >2 or <0.5 were considered differentially expressed.

### Functional enrichment analysis

RNA-seq normalized data (FPKM) were subjected to principal component analysis (PCA) by using an unsupervised approach to observe the whole clustering profile. Gene Ontology (GO, biological processes) and pathway enrichment involved using DAVID (http://david.abcc.ncifcrf.gov/) with the Benjamini and Hochberg FDR to adjust the *p*-value. Significantly enriched GO categories were visualized by using REVIGO (Supek et al., 2011) (http://revigo.irb.hr/). The network of enriched terms was evaluated by using Metascape (Zhou et al., 2019) (http://metascape.org/). To infer the transcription factor regulatory network of this study, we used all 1,665 human transcription factors in the human TFDB 3.0 database (http://bioinfo.life.hust.edu.cn/AnimalTFDB#!/).

### Data availability

All RNA-seq datasets generated in this study have been deposited in Gene Expression Omnibus. Human oocyte, pre-implantation embryo RNA-seq data were obtained from GSE44183 (Xue et al., 2013). The RNA-seq data for normal PN zygotes (n = 22) were downloaded from GSE6548 (Yanez et al., 2016). The list of differentially expressed genes from RNA-seq data for validation were from a previous publication (Suo et al., 2018).

### Statistical analysis

Statistical analysis was performed on original data by using GraphPad Prism 8.0 and Student’s t-test with a significance threshold of *p* < 0.05.

## Results

### Identification of variant in *RGS12* responsible for phenotype of PN arrest of human zygotes

**FIGURE 1:**
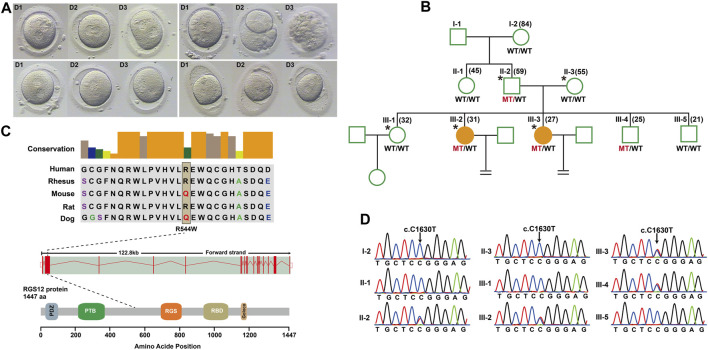
Structure of *RGS12* and sites of pathogenic mutations associated with pronuclear (PN)-arrest zygotes. **(A)** Morphology of zygotes on days 1–3 after IVF. Most zygotes from affected individuals remained at the one-cell stage, and maternal and paternal pronuclei were separated. **(B)** Pedigrees of the family affected by infertility due to arrest at PN zygote stage. Squares denote male family members, circles denote female members, and solid symbols represent affected members. Equal signs indicate infertility. MT, mutant type; WT, wild type. **(C)** Conservation of amino acid residues affected by mutations in different species, structure of *RGS12*, and known domains of the gene product. Exons are red vertical bars, introns are dashed lines, and open rectangles at each end are noncoding exons. **(D)** Sanger sequencing confirmation of *RGS12* variant (c.C1630T) in the family members.

To identify novel PN zygote arrest-specific genes, we recruited a family with multiple infertile individuals who presented recurrent visible PN zygotes with second polar-body emission that failed to complete PN fusion after 24–68 h during IVF (Figure 1A; Supplementary Table S2). The affected individuals had primary infertility with an unknown cause. Initially, the affected individuals presented complete cleavage failure in all 2PN fertilized oocytes after 2 cycles of IVF. Subsequently, the affected individuals underwent 2 cycles of ICSI, with similar outcomes. We performed WES for 2 affected individuals (III-2 and III-3), an unaffected individual (III-1), and their parents (II-2 and II-3) (Figure 1B). Given the pedigree structure, we used an autosomal-dominant inheritance pattern and identified heterozygous, rare, potential pathogenic variants co-segregated with PN zygote arrest. Initially, 13 candidate genes were filtered by WES (Supplementary Table S3), and only the missense variant c. C1630T (minor allele frequency = 0.00018 in ExAC database) resulting in a p. R544W of regulator of G protein signaling-12 (*RGS12*) was confirmed by Sanger sequencing results available for relatives and was characterized by paternal transmission (Figure 1D).

**FIGURE 2:**
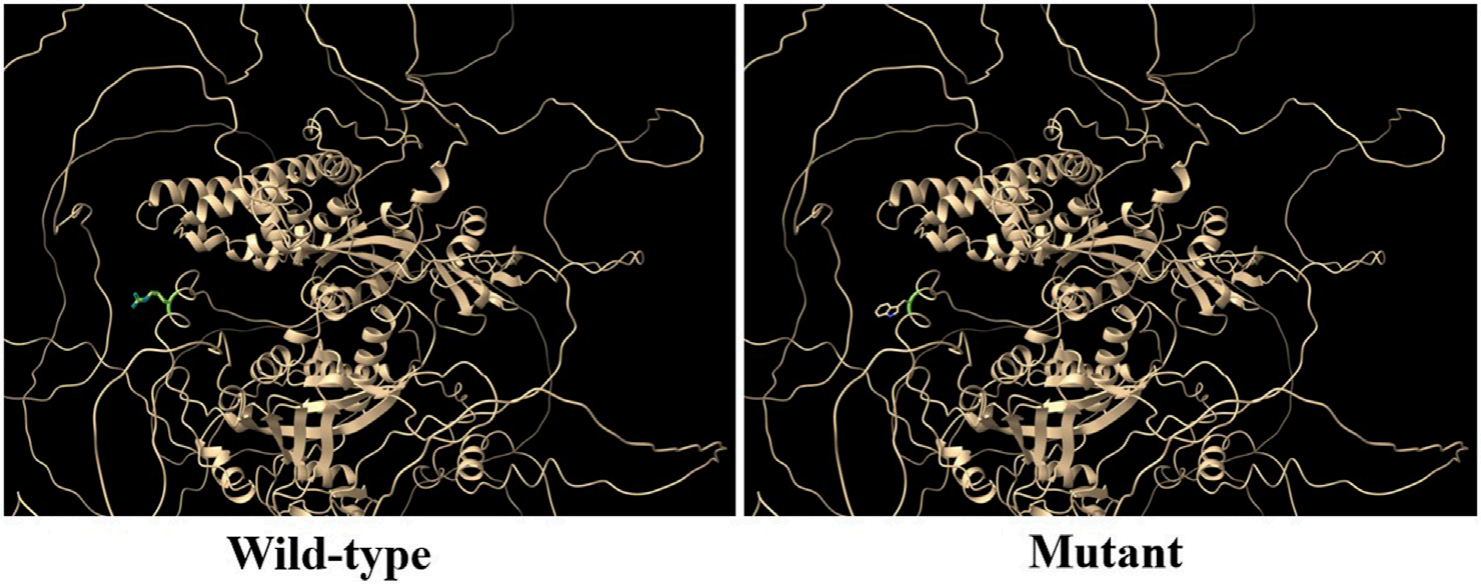
Three-dimensional structural analysis of wild-type and mutant RGS12 protein. Modeled structure of the RGS12 protein was painted by Chimera X software. Green indicated RGS protein structure in wild type, and the blue represent mutated RGS12 protein structure.

*RGS12* is the largest protein in the regulators of the G-protein signaling (RGS) family and is a negative regulator of specific G-protein–coupled receptor (GPCR) signals. *RGS12* p. R544W is located between the RGS and PTB domains and presents a non-conserved pattern between human and mouse (Figure 1C), so it might have a species-specific functional effect. The predicted protein structure of R544W mutant RGS12 was further rendered with ChimeraX (28710774). We observed that the predicted structures of mutational intolerance corresponding to amino acids with inward-facing side chains (Figure 2). Consequently, mutant protein has decreased efficiency of PTB structural domain (PTB), which can bind to N-type calcium channels. RGS12 inhibits Ca^2+^ oscillations through PTB inhibition of G protein activity.

### Identification of the molecular landscape underlying PN-arrest zygotes caused by *RGS12* mutation

**FIGURE 3:**
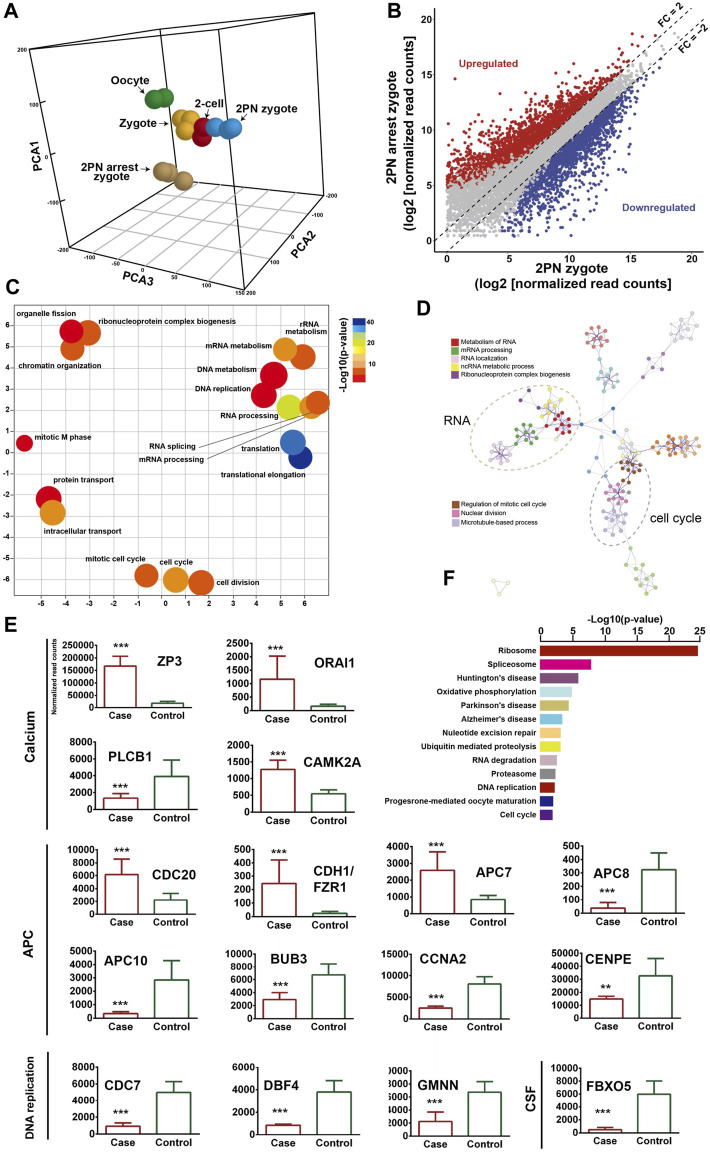
Transcriptome profile of PN arrest zygote by single-cell RNA-sequencing. **(A)** 3-D principal-component analysis (PCA) of the transcriptome of human oocytes, preimplantation embryos and PN-arrest zygotes. Cells of different preimplantation stages form distinct clusters, and PN-arrest zygotes had a specific transcriptome character. **(B)** Scatter plots comparing expression of genes with fold change >2, false discovery rate (FDR) < 0.05. **(C)** REVIGO scatter plot showing the cluster representatives in a 2-D space derived by applying multidimensional scaling to a matrix of semantic similarities for the Gene Ontology (GO) terms (biological processes). **(D)** Metascape enrichment network showing intra- and inter-cluster similarities of enriched GO terms. Cluster annotations are shown in color code. **(E)** Comparative analysis of genes in the Ca^2+^ oscillation–CSF–APC signaling pathway by average RNA-sequencing of normalized read counts for human PN and PN-arrest zygotes. FDR value, Data are mean ± SD. **, *p* < 0.001; ***, *p* < 0.0001. **(F)** Significantly enriched KEGG pathways in PN-arrest zygotes; length of column indicates the–log10 *p*-value.

To describe the molecular landscape underlying the PN-arrest zygote (Figure 3A), we performed single-cell RNA-seq of PN-arrest zygotes (n = 3) from patient III-3 to explore the transcriptional profiles of PN-arrest zygotes by comparison with normal PN zygotes (n = 22, GSE65481) (Yanez et al., 2016). We found significant upregulation of 1,415 genes (fold change >2, *p* < 0.001) and downregulation of 1,545 genes (Figure 3B; Supplementary Table S4). Differentially expressed genes were enriched in the Gene Ontology (GO) terms (biological processes) RNA processing (FDR = 1.13 × 10^−21^), translational elongation (FDR = 1.25 × 10^−16^), intracellular transport (FDR = 1.63 × 10^−15^), and cell cycle (FDR = 4.11 × 10^−13^), so the oocyte-specific transcription and translation machinery is not completely established (Figures 3C, D). Pathway enrichment analysis revealed that differentially expressed genes in PN-arrest zygotes were also mainly involved in RNA processing and translation, such as ribosome (FDR = 6.40 × 10^−22^) and spliceosome (FDR = 1.52 × 10^−5^) (Figure 3F). The transition from oocyte to embryo is driven by a maternal stockpile of mRNA and translational machinery that is “packed” into the oocyte. Furthermore, oocyte activation after fertilization includes changes to oocyte coverings to prevent polyspermy, release of oocyte meiotic arrest, generation of haploid female and male pronuclei, changes in maternal mRNA and protein populations, and cytoskeletal rearrangements. Our transcriptional prolife results implied that the PN-arrest zygotes had properties of failure of complete oocyte activation after fertilization.

### Mutant *RGS12* affects Ca^2+^ oscillations during oocyte activation after fertilization

Oocyte activation events present different Ca^2+^ requirements: 1) for cortical granules and blocking polyspermy; 2) inducing the resumption of meiosis including second meiotic polar body extrusion and initiating recruitment of maternal mRNAs; and 3) promoting pronuclear formation and initiation of embryonic mitosis. On oocyte activation, after PN formation, Ca^2+^ signaling continues to play a role in PN fusion and DNA synthesis for initiating embryonic mitosis during the oocyte-to-embryo transition.

Mature oocytes await fertilization while arrested at MII, which is maintained by the maturation promoting factor (MPF) consisting of cyclin B1/CDK1 subunits. Cytostatic factor (CSF) mediates MPF stabilization by inhibiting the anaphase-promoting complex (APC), which would otherwise destroy cyclin B. Fertilization breaks the MII arrest via cytoplasmic Ca^2+^ oscillation and triggers the APC, which mediates the degradation of cyclin B and thus inactivation of MPF. To confirm the spontaneous and abnormal Ca^2+^ oscillations, we traced the transcriptional change in genes that participate in vital processes from Ca^2+^ oscillation, CSF and APC (Figure 3E).

PN-arrest zygotes showed 9.54-fold increased *ZP3* expression, which indicates sustained *ZP3*-evoked Ca^2+^ entry by Ca^2+^ influx and activation of G protein (Patel, 2004). ZP3-mediated calcium in-flow is important for fertilization, but abnormal calcium in-flow may be an important manifestation of PN arrest zygotes. Furthermore, plasma membrane Ca^2+^ channel *ORAI1* mediates Ca^2+^ influx of oocytes after fertilization. In PN-arrest zygotes, *ORAI1* showed 7.2-fold upregulation, which further confirmed the Ca^2+^ influx. These results indicate a spontaneous and abnormal Ca^2+^ oscillation in PN-arrest zygotes.

Next, we explored further evidence to support spontaneous and abnormal Ca^2+^ oscillation and its effects. *CAMKII* (*CAMK2A*) links Ca^2+^ oscillations and inactivates the MPF as well as translation and degradation of maternal mRNAs. PN-arrest zygotes showed upregulated *CAMK2A*, which implies the existence of prolonged Ca^2+^ oscillations. *CaMKII* activation by Ca^2+^ oscillations leads to activation of the APC by inhibiting CSF activity, which suppresses APC via *EMI1* working with proto-oncogene, serine/threonine kinase (MOS). Because of abnormal Ca^2+^ oscillations, *EMI1* (*FBXO5*) was significantly downregulated (12.25-fold) in PN-arrest zygotes, which indicates the lower CSF level and possible high APC level.

Activation of APC is regulated by 2 activators, *CDC20* and *CDH1*. *CDC20* and *CDH1* expression was 2.7- and 10.3-fold increased, respectively, in PN-arrest zygotes. *CDC20* and *CDH1* bind to APC7, whose level was also increased in PN-arrest zygotes. The mitotic checkpoint complex (MCC), composed of *CDC20, MAD2, BUBR1, CENPE* and *BUB3*, acts as an APC inhibitor (Chao et al., 2012), but we found downregulated *BUB3* and *CENPE* in PN-arrest zygotes. Downregulation of *EMI1* and *BUB3* and upregulation of *CDC20, CDH1,* and *APC7* implied continually increased APC level in PN-arrest zygotes.

*APC10* and cyclin A2 (*CCNA2*) were downregulated in PN-arrest zygotes, with no change in *CCNB1* expression. We also found downregulation of *GMNN* (geminin), an APC substrate and essential for regulation of DNA replication for zygotes. In PN-arrest zygotes, *CDC7/DBF4* expression was decreased ∼ five-fold. Therefore, continuous APC disrupted nuclear envelope breakdown (NEBD) and DNA replication in PN-arrest zygotes.

Increased expression of ZP3 and Orai1 in PN arrest zygotes may lead to disturbances in calcium inward flow and promote APC activity. Here we speculate that spontaneous and abnormal Ca^2+^ oscillation increased APC level by the mutant *RGS12*, thus leading to defective NEBD and DNA replication after 24–68 h in IVF trials. The trigger for the oocyte-to-embryo transition is oocyte activation. Therefore, our evidence supports that the PN arrest is due to spontaneous and abnormal Ca^2+^ oscillation causing prolonged APC activation. Hence, a precise pattern of Ca^2+^ oscillations after fertilization should be evaluated for further treating optimal oocyte activation.

### Validation of Ca^2+^ oscillation–CSF–APC signaling in PN arrest zygotes

**FIGURE 4:**
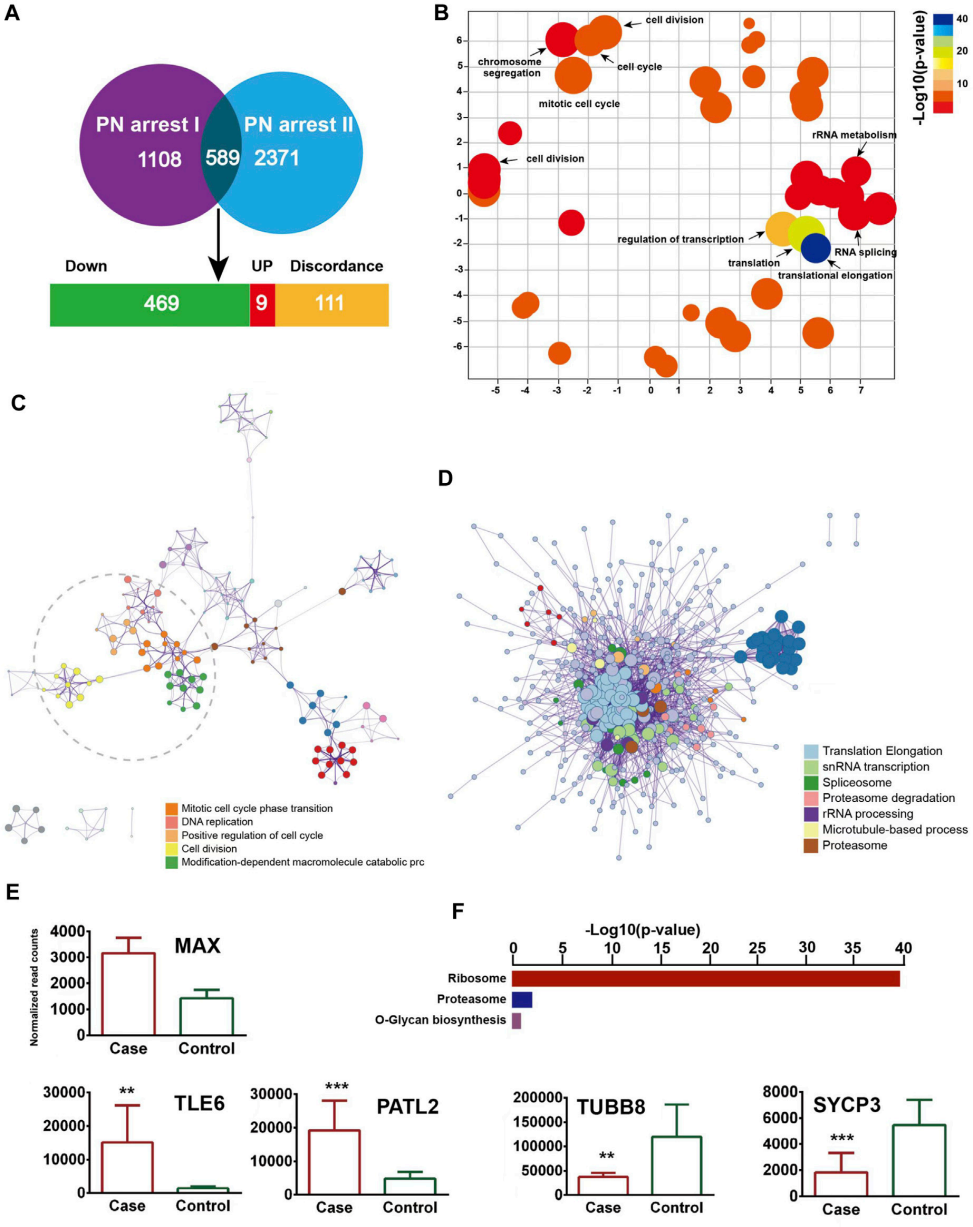
Validation of transcriptome profile in other PN-arrest zygotes. **(A)** Venn diagram shows overlapped differentially expressed genes between previous study by Suo et al. (2018) (PN arrest I) and our study (PN arrest II) for PN arrest groups each compared with their control group. **(B)** REVIGO scatterplot summarizes the overrepresented GO terms (biological processes) for representative subsets of terms. **(C)** Metascape enrichment network of the intra-cluster and inter-cluster similarities of enriched GO terms. **(D)** Metascape interactome network formed by all GO terms, confirming the defective RNA processing and translation in both PN-arrest zygote groups. **(E)** Relative expression of particularly interesting DEGs. FDR value, Data are mean ± SD. **, *p* < 0.001; ***, *p* < 0.0001. **(F)** Significantly enriched KEGG pathways shared in PN-arrest zygotes.

We identified 589 common genes enriched in the GO terms translational elongation (FDR = 5.88 × 10^−34^) and translation (FDR = 2.45 × 10^−17^) and confirmed the incomplete oocyte activation. The key components of Ca^2+^ oscillation, CSF and APC signaling, *EMI1, CCNA2, CDC7/DBF4,* and *GMNN* were also identified (Figures 4A–D). To investigate the master regulators and construct the transcriptional regulatory network in the PN-arrest zygotes, we used the ARACNe method to analyze transcription factors. Only the transcription factor *MAX* was upregulated (Figure 4E), so *MYC-MAX* may play a critical role in the cell cycle entry of PN-arrest zygotes.

We found upregulation of 2- to 4-cell arrest-specific genes *TLE6* and *PATL2*; hence, the *RGS12* mutation caused earlier embryonic development arrest (Figure 4E). Furthermore, *SYCP3* and *TUBB8* were downregulated, which indicates defective cytoskeletal rearrangements. We did not find a disruption of zygotic arrest 1 (*ZAR1*), a oocyte-specific maternal-effect gene for mouse oocyte-to-embryo transition (Wu et al., 2003).

## Discussion

In this study, we used WES to explore mutations affecting familial individuals with recurrent PN arrest during IVF and ICSI cycles. We identified a heterozygous pathogenic variant in RGS12 that was responsible for the phenotype. Moreover, single-cell RNA-seq identified proximal genes involved in Ca^2+^ oscillation–CSF–APC signaling. The findings indicate that abnormal spontaneous Ca^2+^ oscillations led to oocytes with prolonged low CSF level and high APC level, which resulted in defective NEBD and DNA replication.

Maternal genes have a critical effect in the earliest stages of embryonic development. *ZAR1* was first identified as an oocyte-specific maternal-effect gene that functions at the oocyte-to-embryo transition in mice (Wu et al., 2003), Novel synonymous variation (c.516C>T) and intron variation (c.964-55A>T) of *ZAR1* were identified in individuals with recurrent uncleaved zygotes in IVF (Tian et al., 2020). Homozygous mutations in *BTG4* caused zygotic cleavage failure in 4 independent affected females with infertility of unknown cause (Zheng et al., 2020). *BTG4/CCR4-NOT*–induced mRNA deadenylation is involved in regulating maternal mRNA stability (Sha et al., 2020). However, nothing is known about the genetic cause of the phenotype of human PN-arrest zygotes from familial individuals.

Here we describe a rare family with multiple infertile female members with phenotypes of human PN-arrest zygotes. Use of WES revealed that the affected members carried a paternally originated autosomal-dominant mutation (p.R544W) in *RGS12*, a member of the family of regulators of G-protein signaling. *RGS12* variants have been considered the most promising candidates in multiple families affected by bipolar disorder identified by WES (Forstner et al., 2020). To the best of our knowledge, this is the first report to describe a variant in the *RGS12* responsible for female infertility characterized by arrest at the PN stage during multiple IVF. The genetic basis for infertility characterized by abnormalities in human oocyte development and early embryogenesis (2- to 4-cell) has been described (Alazami et al., 2015; Feng et al., 2016; Xu et al., 2016). A variant in *RGS12* extends the genetic causes of infertility. Therefore, our findings will facilitate genetic diagnoses for *RGS12* mutation in identifying patients with infertility who are undergoing IVF and ICSI.

**FIGURE 5:**
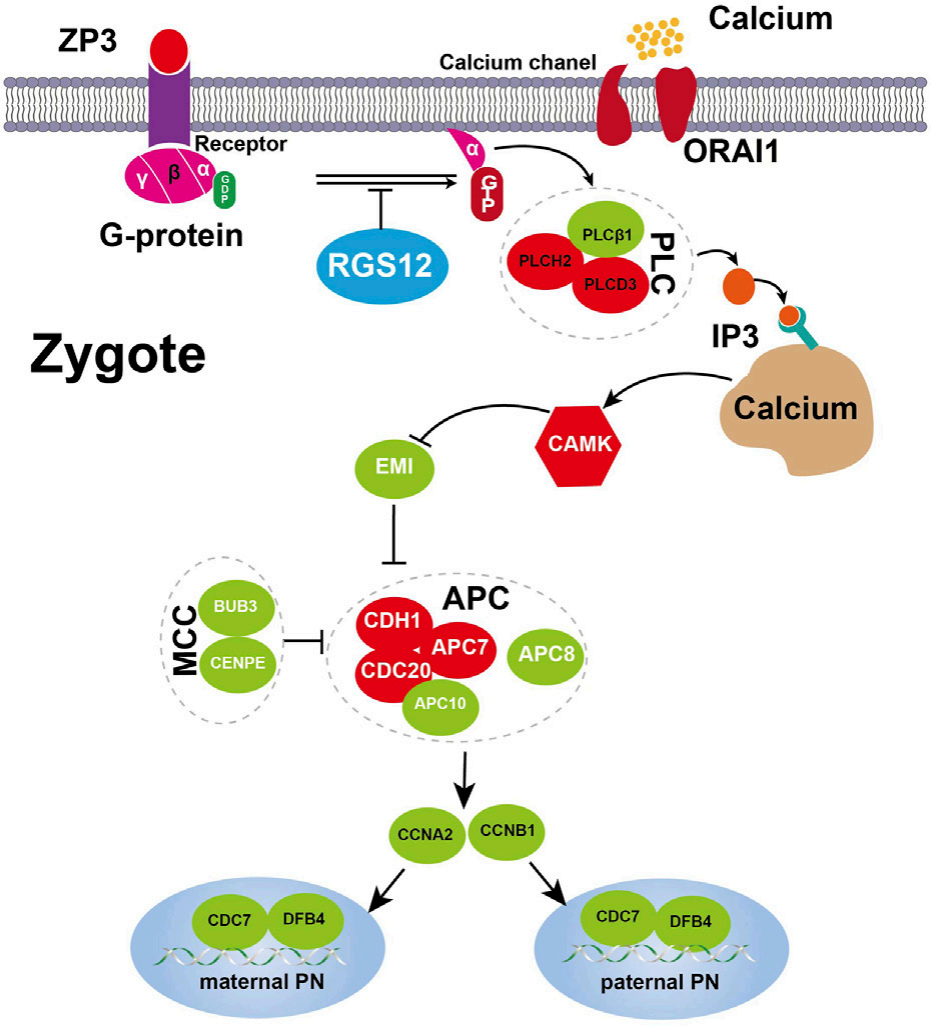
Expression changes of key genes involved Ca^2+^ oscillation–CSF–APC signaling. A proposed model for the *RGS12*-mutant oocyte with spontaneous and abnormal Ca^2+^ oscillations leading to prolonged low CSF and high APC level resulting in defective nuclear envelope breakdown and DNA replication. The key component genes are represented by green (downregulation) and red (upregulation), respectively. Detailed explanations are provided in the text.

*ZAR1* regulates mRNA translation during the oocyte-to-embryo transition (Tian et al., 2020). *BTG4* variants impair the decay of maternal mRNA in zygotes of the affected individual with zygotic cleavage failure (Zheng et al., 2020). Unlike *ZAR1* and *BTG4*, which affect maternal mRNA translation, *RGS12* plays an important role in Ca^2+^ oscillations in osteoblasts, neurons and other cell types (Schiff et al., 2000; Richman et al., 2005; Li et al., 2019). Therefore *RGS12* controls Ca^2+^ oscillations, which provides an important spatially restricted Ca^2+^ signal required for complete oocyte activation after fertilization and triggers CSF–APC signaling to switch from meiosis to mitotis. Our single-cell transcriptome sequencing data revealed unique features in translation, RNA processing and cell-cycle impairments of failure of complete oocyte activation and uncovered the Ca^2+^ oscillation–CSF–APC signaling pathway by which mutant *RGS12* exerts its maternal effect on PN arrest. The genes involved in PN arrest of the Ca^2+^ oscillation–CSF–APC signaling pathway were validated in other studies (Suo et al., 2018). The partially activated oocytes not progressing further and becoming arrested again in the PN stage were described as a new MII arrest (Kubiak, 1989). The key genes underlying the PN-arrest zygote (Figure 5) improve our understanding of why and how *RGS12* mutation causes the phenotype.

Our validated single-cell transcriptional data support that a loss-of-function effect of *RGS12* p. R544W causes spontaneous and abnormal Ca^2+^ oscillations in zygotes with 2PN arrest after fertilization. Our findings could explain why artificial oocyte activation using the A23187 Ca^2+^ ionophore improved embryo development only to the cleavage stage at a limited rate (one of 4 couples) (Darwish and Magdi, 2015). Spontaneous and abnormal Ca^2+^ oscillations in zygotes with 2PN arrest also raise the possibility of developing antagonists to block the excessive free Ca^2+^, such as Ca^2+^ chelators BAPTA and EGTA.

## Conclusion

We have identified an *RGS12* variant as the potential cause of female infertility characterized by arrest at the PN stage during multiple IVF and ICSI. This gene should be further screened in individuals with infertility caused by arrest at the PN stage during IVF and ICSI. These findings expand our knowledge of the genetic basis of human early embryonic arrest and provide the basis for genetic diagnoses of clinically infertile individuals with this phenotype.

## Data Availability

Sequencing data have been deposited in public, open access repository of the Genome Sequence Archive for Human (http://bigd.big.ac.cn/gsa-human/) with the accession no. HRA006945.

## Abbreviations

APC, anaphase-promoting complex; BUB3, mitotic checkpoint protein; CaMKII, Calcium/calmodulin-dependent protein kinase II; CCNA2, cyclin A2; CDC, cell division cycle; CDH1, cadherin 1; CENPE, centromere protein E; CSF, cytostatic factor; EMI1, F-box protein 5; GMNN, geminin DNA replication inhibitor; ICSI, intracytoplasmic sperm injection; IVF, *in vitro* fertilization; ORAI1, calcium release-activated calcium modulator 1; PN, pronuclear; RGS12, regulator of G protein signaling 12.
