# Supplementary material for: A case report of a family with developmental arrest of human prokaryotic stage zygote
Source: Front Cell Dev Biol. 2024 Mar 28;12:1280797. doi: 10.3389/fcell.2024.1280797 (PMC11006971; doi:10.3389/fcell.2024.1280797)
Supplement: Supplementary file 2 [file Table1.DOCX]

| **Case** | **Aage**  **(years)** | **AFC** | **Infertility category** | **Duration of infertility** | **Chromosome** | **BMI**  **(kg/m2)** | **Basal FSH**  **(IU/L)** | **Basal E2**  **(pg/L)** | **Basal P**  **(ng/L)** | **Basal LH**  **(IU/L)** | **Basal PRL(ng/L)** | **Basal T(ng/L)** | **AMH**  **(ng/mL)** |
| --- | --- | --- | --- | --- | --- | --- | --- | --- | --- | --- | --- | --- | --- |
| **Case III-3** | 25 | 20 | Primary | 2 years | 46.XX | 18.92 | 7.080 | 88.690 | 0.000 | 3.170 | 31.750 | 0.665 | 1.26 |
| **Case II-2** | 29 | >24 | Primary | 2 years | 46.XX | 18.82 | 7.260 | 86.000 | 4.470 | 2.870 | 16.300 | 2.090 | 1.54 |

**Supplementary Table 1. Clinical basal data for patients**
