# Supplementary material for: A case report of a family with developmental arrest of human prokaryotic stage zygote
Source: Front Cell Dev Biol. 2024 Mar 28;12:1280797. doi: 10.3389/fcell.2024.1280797 (PMC11006971; doi:10.3389/fcell.2024.1280797)
Supplement: Supplementary file 3 [file Table2.DOCX]

| **Patients** | **Clinical cycles** | **Patient with PN-arrest zygotes** | | | | | | | |
| --- | --- | --- | --- | --- | --- | --- | --- | --- | --- |
|  |  | **Ovarian stimulation** | **ART method** | **Oocyte** | | | | **Fertilization** | |
|  |  |  |  | **Total** | **MII** | **MI** | **GV** | **2PN** | **PN-arrest** |
| **III-3** | **1st** | long agonist protocol | IVF | 10 | 10 | 0 | 0 | 9 | 7 |
|  | **2nd** | long agonist protocol | IVF+ICSI | 18 | 18 | 0 | 0 | 14 | 11 |
|  | **3rd** | Mild stimulation protocol | IVF | 10 | 8 | 2 | 0 | 8 | 6 |
| **III-2** | **1st** | long agonist protocol | IVF | 19 | 14 | 2 | 3 | 13 | 13 |
|  | **2nd** | Mild stimulation protocol | IVF+ICSI | 12 | 12 | 0 | 0 | 12 | 12 |

**Supplementary Table 2. Embryonic development consequence of the patients with PN arrest during IVF.**

IVF*: in vitro* fertilization; ICSI: intracytoplasmic sperm injection
